# Liver Fat Measured by MR Spectroscopy: Estimate of Imprecision and Relationship with Serum Glycerol, Caeruloplasmin and Non-Esterified Fatty Acids

**DOI:** 10.3390/ijms17071089

**Published:** 2016-07-08

**Authors:** Michael France, See Kwok, Handrean Soran, Steve Williams, Jan Hoong Ho, Safwaan Adam, Dexter Canoy, Yifen Liu, Paul N. Durrington

**Affiliations:** 1 Department of Clinical Biochemistry, Cobbett House, Central Manchester Foundation Trust, Oxford Road, Manchester M13 9WL, UK; 2 Cardiovascular Trials Unit, The Old St Mary’s Hospital, Hathersage Road, Oxford Road, Manchester M13 9WL, UK; sk7@doctors.org.uk; 3 Department of Medicine, Central Manchester Foundation Trust, Oxford Road, Manchester M13 9WL, UK; hsoran@aol.com; 4 Department of Imaging Science, University of Manchester, Oxford Road, Manchester M13 9PT, UK; steve.williams@manchester.ac.uk (S.W.); s.adam@doctors.org.uk (S.A.); 5 Cancer Epidemiology Unit, University of Oxford, Richard Doll Building, Roosevelt Drive, Oxford OX3 7LF, UK; dexter.canoy@ceu.ox.ac.uk; 6 School of Biomedicine, 3rd floor, Core Technology Facility, 46 Grafton Street, Manchester M13 9NT, UK; yifen.liu@manchester.ac.uk (Y.L.); paul.durrington@manchester.ac.uk (P.N.D.)

**Keywords:** fatty liver, NEFA, glycerol, lipolysis, insulin, magnetic resonance spectroscopy

## Abstract

Magnetic resonance spectroscopy (MRS) is a non-invasive method for quantitative estimation of liver fat. Knowledge of its imprecision, which comprises biological variability and measurement error, is required to design therapeutic trials with measurement of change. The role of adipocyte lipolysis in ectopic fat accumulation remains unclear. We examined the relationship between liver fat content and indices of lipolysis, and determine whether lipolysis reflects insulin resistance or metabolic liver disease. Imprecision of measurement of liver fat was estimated from duplicate measurements by MRS at one month intervals. Patients provided fasting blood samples and we examined the correlation of liver fat with indices of insulin resistance, lipolysis and metabolic liver disease using Kendall Tau statistics. The coefficient of variation of liver fat content was 14.8%. Liver fat was positively related to serum insulin (T = 0.48, *p* = 0.042), homeostasis model assessment (HOMA)-B% (T = −0.48, *p* = 0.042), and body mass index (BMI) (T = 0.59, *p* = 0.012); and inversely related to HOMA-S% (T = −0.48, *p* = 0.042), serum glycerol (T = −0.59, *p* = 0.014), and serum caeruloplasmin (T = 0.055, *p* = 0.047). Our estimate of total variability in liver fat content (14.8%) is nearly twice that of the reported procedural variability (8.5%). We found that liver fat content was significantly inversely related to serum glycerol but not to non-esterified fatty acids (NEFA), suggesting progressive suppression of lipolysis. Reduction of caeruloplasmin with increasing liver fat may be a consequence or a cause of hepatic steatosis.

## 1. Introduction

Nonalcoholic fatty liver disease (NAFLD) is associated with the histological finding of hepatic steatosis or steatohepatitis and has a number of causes [1,2,3,4]. Steatosis is defined as a liver fat content of greater than 5% [3,5], and may be detected by ultrasonography in patients investigated for abnormal serum transaminase levels. It is a common finding in patients with hypertriglyceridaemia and is frequently accompanied by insulin resistance and other features of metabolic syndrome. Although the condition is usually benign, 10% of patients do progress to nonalcoholic steatohepatitis (NASH), of whom 25% may proceed to cirrhosis [2].

Magnetic resonance spectroscopy (MRS) is a non-invasive and effective method in assessment of hepatic fat accumulation with high diagnostic accuracy and correspondence with histopathologic grade being demonstrated [6]. Imprecision in the measurement of liver fat content by MRS comprises biological variability and measurement error. It is an important consideration in the design of therapeutic trials aiming to measure change in liver fat content. We estimated imprecision from duplicate measurements at an interval of one month and compared our estimate with variability reported after immediate repetition of MRS in 10 individuals with similar characteristics. Although it has been suggested that accumulation of liver fat in metabolic syndrome is driven by increased hepatic fatty acid delivery due to adipocyte insulin resistance [7], raised levels of non-esterified fatty acids (NEFA) are not always found in hepatic steatosis [8]. We investigated the relationship between liver fat and indices of lipolysis and metabolic liver disease as these have the potential to influence biological variability.

## 2. Results

**Figure 1:**
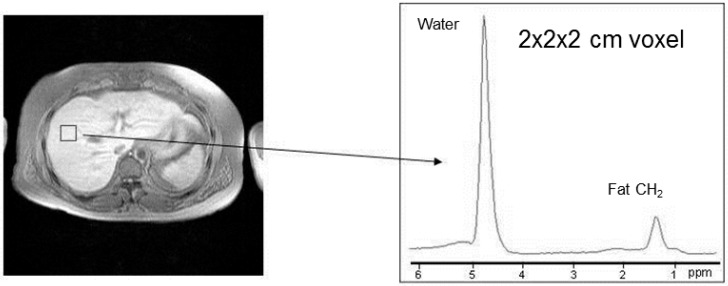
Transverse magnetic resonance (MR) image through the abdomen and localised MR spectrum recorded from the 2 × 2 × 2 cm voxel placed over the liver. The frequency axis of the spectrum is expressed in parts per million (ppm).

The distribution of differences between duplicate liver fat measurements was sufficiently normal (Shapiro–Wilk 0.7612) to calculate imprecision from the differences, with coefficient of variation 14.8%. The median body mass index (BMI) was 30.8 kg/m^2^ (range 20.2–40.4) with 2 patients having a BMI <25 kg/m^2^. MR image of the abdomen and a spectrum from the liver from one patient are shown in Figure 1. Both water and triglyceride signals are visible at high signal-to-noise. Median liver fat content was 44 g·kg^−1^ water (range, 10–332). Triglycerides were greater than 1.7 mmol·L^−1^ in 10 out of 11 patients. Hyperinsulinaemia was present in all patients although only one had a fasting plasma glucose in the impaired glucose tolerance range >6.1 mmol·L^−1^ and one in the diabetes range at 7.5 mmol·L^−1^. Nine patients had supra-normal β cell function with (homeostasis model assessment (HOMA)-B% >100%) and all patients had impaired insulin sensitivity (HOMA-S% < 100%, median 43.9% and range 13.3–91.9). Table 1 shows the correlation of metabolic parameters related to insulin resistance, alcohol intake, ferritin, iron studies, α-1 antitrypsin (A1AT), and caeruloplasmin with the average of the two liver fat measurements.

**Figure 2:**
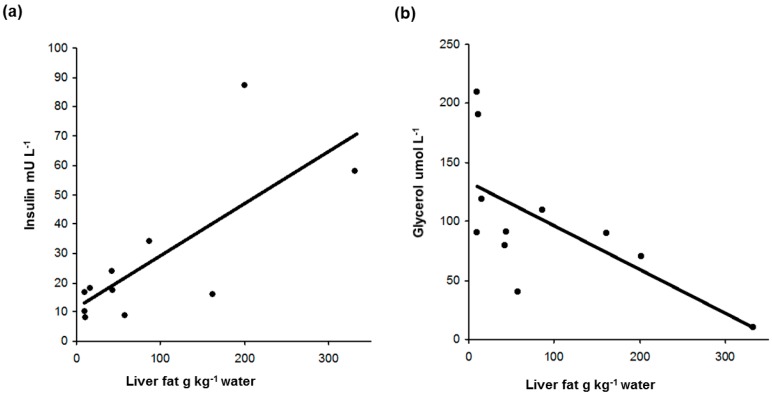

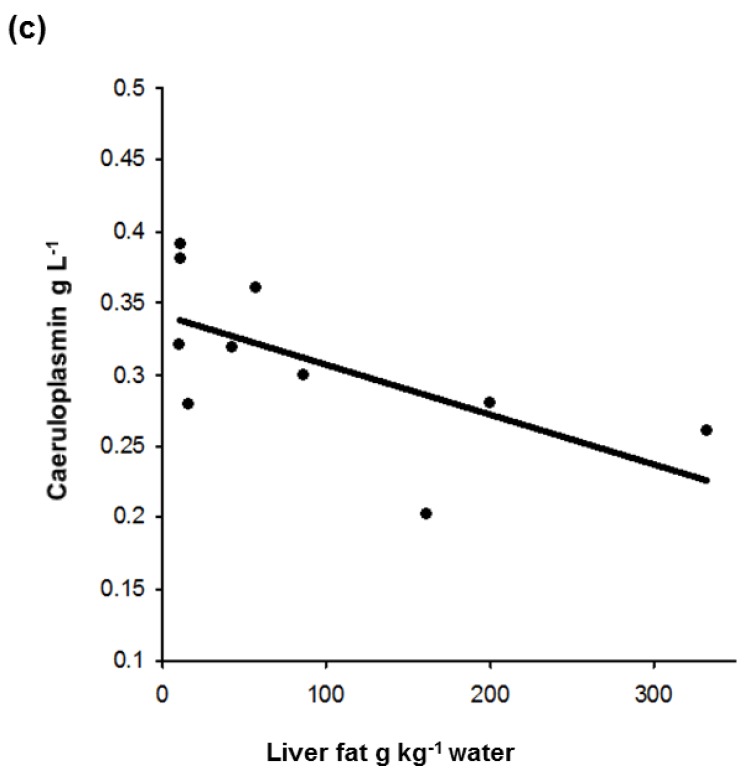
Relationship of insulin (**a**); glycerol (**b**); and caeruloplasmin (**c**) with liver fat content.

The correlations between insulin, glycerol, and caeruloplasmin and liver fat are illustrated in Figure 2. Insulin (Figure 2a) and HOMA-B% were positively related to liver fat whereas HOMA-S% was inversely related (these are identical because insulin concentration is a component of all three and the ranked pairs of observations in this small series, by chance, are the same). NEFA and glycerol (Figure 2b) were inversely related to liver fat, but this inverse correlation was only significant for glycerol. The median NEFA was 302 umol·L^−1^ with range 138–491 umol·L^−1^, and all were in the lower half of the reference range (130–1050 umol·L^−1^). Glycerol (reference range 27–37 umol·L^−1^) had a wider range of 10–210 umol·L^−1^ and median 90 umol·L^−1^ reflecting suppression with high liver fat and high levels with low liver fat. Glucose, triglycerides, alcohol intake, ferritin, iron, % iron binding capacity, and A1AT were not related to liver fat but caeruloplasmin (Figure 2c) was inversely related. One patient had a caeruloplasmin level below the lower reference interval but Wilson’s disease was excluded by follow-up studies. There were no differences in liver fat content between the following groups: “untreated with statins or fibrates”, “statin monotherapy” or “fibrate monotherapy” (*p* = 0.5), or between groups either taking or not taking Omacor (*p* = 0.2).

## 3. Discussion

Our data on repeat MRS at one month intervals showed a coefficient of variation of 14.8%, which is higher than the coefficient of variation of 8.5% observed between repeat MRS taken at 10 min intervals [5]. This difference likely reflects the technical challenge of repositioning the subject and reproducing conditions of the scan after one month. This would also have been contributed by alterations in hepatic adiposity in the subjects during the time period. It is important to take account of the overall imprecision of repeated measurements of liver fat in the design of therapeutic trials. Duplicate measurements improved the estimate of liver fat content in this study.

In this group of patients, we found no evidence of increased lipolysis despite increasing insulin resistance with increasing liver fat content. Higher liver fat content was significantly associated with lower serum glycerol but not NEFA. Glycerol was suppressed to quite low levels with increasing liver fat. In fact, in the subject with liver fat >30 g·kg^−1^ water, glycerol was almost completely suppressed. NEFA levels are in the lower half of the reference range with a downward trend as liver fat increased. It is interesting that the relationship between liver fat and glycerol is stronger than that of NEFA. Glycerol is regarded as a better reflection of adipocyte lipolysis than NEFA because, unlike NEFA, once released it cannot be taken up by the adipocyte again [9]. Our findings, therefore, do not accord with the hypothesis that increased delivery of NEFA secondary to adipocyte insulin resistance causes ectopic hepatic fat accumulation [1]. Indeed, the role of increased lipolysis in ectopic fat accumulation has been questioned in a previous study [8], with an alternative mechanism of diversion of chylomicron fatty acid to ectopic storage sites due to dysfunctional adipose tissue being proposed. It is suggested that this occurs with down regulation of NEFA trafficking and preservation of serum NEFA. Furthermore, obese subjects have been shown to have a reduction in NEFA release per unit of adipose tissue with no difference in NEFA levels compared with lean controls, and have reduced adipose tissue lipolysis [10].

The range of liver fat found in our subjects was similar to that found in the Dallas Heart Study [5]. Despite fatty liver having been reported by ultrasonography, 6 patients had a level of liver fat below the 95th centile of 5.6 g·kg^−1^ water cut-off established in a sub-set of the Dallas Heart Study’s population without risk factors for fatty liver and normal serum transaminase levels. This may reflect the qualitative nature of hepatic ultrasound assessment of liver fat but may also reflect variability in liver fat content, particularly at near normal levels. Our subjects were not required to fast for the MRS because this has been shown not to contribute to variability [5].

The observed negative relationship of caeruloplasmin with hepatic steatosis is unexplained. Transferrin, A1AT, and caeruloplasmin are acute phase proteins, all of which increases with inflammation. Decreasing caeruloplasmin is, therefore, unlikely to reflect the inflammatory component of steatohepatitis. The decrease in caeruloplasmin could reflect reduced secretion of holoprotein due to failure to incorporate copper, an occurrence similar to that in Wilson’s disease, decreased synthesis, and increased catabolism. Our results are consistent with a recent report demonstrating reduced hepatic copper related to the severity of steatosis in patients with NAFLD [11]. Furthermore, a reduction in caeruloplasmin measured as copper oxidase activity has been noted in alcoholic liver disease implying reduced incorporation of copper into caeruloplasmin [12]. The role of hepatic copper in steatosis remains undefined. One of our patients had a false positive caeruloplasmin test for Wilson’s disease with a value below the lower limit of the reference range, suggesting a potential need to adjust the cut-off in the context of NAFLD.

## 4. Materials and Methods

### 4.1. Subjects

We recruited eleven patients (10 males and 1 female) attending the lipid outpatient clinic who had elevated serum alanine transaminase (ALT) levels and established hepatic steatosis demonstrated by ultrasonography. Their baseline characteristics are shown in Table 2.

A diagnosis of fatty liver was made by exclusion. The presence of biliary obstruction or other structural abnormalities were excluded on ultrasonography. Autoimmune liver disease, chronic hepatitis and metabolic liver disease were excluded by the presence of normal immunoglobulin levels, absence of autoantibody markers, negative serological tests for hepatitis B and C, and measurements of serum ferritin, iron saturation, caeruloplasmin, and α-1 antitrypsin (A1AT). No patient had any clinical manifestations of Wilson’s disease. Excess alcohol consumption (greater than 24 units per week for men and 14 units per week for women) was excluded on detailed history. Patients treated with hypoglycaemic agents were excluded. The study was restricted to subjects with ALT levels less than three times the upper limit of normal (120 U·L^−1^). All patients were following a cardioprotective diet and were also provided with advice regarding recommended levels of physical activity which comprises 150 min of moderate intensity aerobic physical activity or 75 min of high intensity aerobic physical activity per week in combination with muscle-strengthening activities for at least 2 days a week. Drug treatment was unchanged for six months prior to and during the study. Daily drug treatment was targeted at treatment of combined dyslipidaemia and consisted of no treatment (*N* = 3), statin monotherapy (Simvastatin 10 mg o.d., Simvastatin 40 mg o.d. and Atorvastatin 80 mg o.d.) (*N* = 3), statin in combination with Omacor (Atorvastatin 80 mg o.d. with Omacor 2 g per day) (*N* = 1), fibrate monotherapy (Fenofibrate 160 mg o.d. and 200 mg o.d.) (*N* = 2), fibrate in combination with Omacor (Fenofibrate 267 mg o.d. with Omacor 2 g per day) (*N* = 1), and Omacor monotherapy (4 g per day) (*N* = 1). None of the patients were on thyroxine, β blockers, thiazolidinediones or thiazide diuretics. All patients provided blood samples in clinic following a minimum of 12 h fasting and had their height and weight measured, which was used to calculate their body mass index (BMI) as weight (kg) × height (m^−2^).

### 4.2. Laboratory Methods

Serum total cholesterol, high density lipoprotein cholesterol (HDL), triglycerides, iron, total iron binding capacity (TIBC), % iron saturation of TIBC, and fluoride oxalate plasma glucose were measured routinely using the standard laboratory protocols of the Department of Clinical Biochemistry at the Central Manchester University Hospital NHS Foundation Trust (CMFT) using a Roche Modular P Analyzer. Serum caeruloplasmin was measured by a nephelometric assay on the Beckman Array Analyser using a Beckman calibrator. Serum ferritin was measured using the standard laboratory protocol of the Department of Clinical Haematology at CMFT on a Beckman Access Analyser with reagents supplied by Beckman Coulter. Serum glycerol was measured using Sigma Aldrich GPO PAP reagents and serum NEFA were measured using Wako NEFA C ACS-ACOD reagents (Wako Chemicals GmbH, Neuss, Germany) on a Roche Cobas Mira analyzer (Roche, Basel, Switzerland). Serum insulin was measured by an “in house” method using a polyclonal anti-porcine insulin, raised in guinea-pig obtained from Diagnostics Scotland, Carluke, Scotland, UK and using ^125^I labeled Insulin (DSL-1620, 185kBq, DSL Ltd.) obtained through Oxford Bio-Innovation Ltd., Bicester, UK. HOMA-S% and HOMA-B% were calculated using the Oxford University Calculator HOMA2 2004 [13].

### 4.3. Estimation of Liver Fat

Two MRS of the liver were performed, at one month intervals, in each patient using a Philips 1.5 Tesla *Achieva* MR scanner (Amsterdam, The Netherlands). After subjects were positioned to allow access to an area free of blood vessels, fully relaxed (repetition time, TR = 6 s) and localised ^1^H MR spectra were obtained from a 2 × 2 × 2 cm volume using PRESS localization without water suppression (echo time, TE = 23 ms, 32 averages). T_2_ relaxation times (the time constant for decay of transverse components of magnetisation (M_xy_)) for water and fat were estimated from a series of 5 spectra recorded in each session (8 averages, TR = 1600) at TE values of 23, 50, 100, 150, and 200 ms. Analysis of the spectra was performed using the AMARES routine in the jMRUI deconvolution software (MRUI consortium) [14], which provided a ratio of intracellular triglyceride to water. The ratio was corrected for T_2_ relaxation time differences between water and fat [15,16]. In order to provide consistency between serial scans, the second scan performed after a one month interval was obtained in a similar position with the aid of the first scan. The MRS procedure was well tolerated with only one patient experiencing claustrophobia.

### 4.4. Statistical Methods

The standard deviation of MRS estimates of liver fat was calculated from the differences between the two scans as √ [∑ (differences^2^)/22]. The normality of the distribution of differences was assessed using the Shapiro–Wilk W test. All other data are expressed as median (range) because of their non-parametric distribution. Correlation between variables was calculated as the Kendall Tau rank statistic with a 2 tailed probability of <0.05 being regarded as significant. The Kruskal Wallace one way analysis of variance test was used to assess the differences in liver fat content between 3 groups defined by drug treatment as: “no statin or fibrate treatment”, “statin monotherapy”, and “fibrate monotherapy”, which were mutually exclusive, and between 2 groups defined as “Omacor treated” or “not Omacor treated”.

The study was designed to estimate the variability of sequential measures of liver fat to inform power calculation for future studies. The estimate was considered sufficiently robust after 11 patients, after review by our statistician.

## 5. Main Messages

Variability of repeat scans performed one month apart is nearly twice that observed with immediate repetition, and should be taken into account in the design of interventional trials with liver fat content as the endpoint. Glycerol is inversely related to liver fat content suggesting down regulation of fatty acid trafficking consistent with the new paradigm for the pathogenesis of fatty liver. Caeruloplasmin is inversely related to liver fat content, which is as yet unexplained.

## Figures and Tables

**Table 1:** Kendall Tau rank correlation between liver fat and metabolic parameters.

Measurement	Tau	*p* Value
BMI kg·m^−2^	0.59	0.012
NEFA umol·L^−1^	−0.22	0.3
Glycerol umol·L^−1^	−0.59	0.014
Glucose mmol·L^−1^	0.13	0.5
Insulin mU·L^−1^	0.48	0.042
HOMA-S%	−0.48	0.042
HOMA-B%	0.48	0.042
Triglyceride mmol·L^−1^	0.37	0.1
Caeruloplasmin g·L^−1^	−0.55	0.047
Iron umol·L^−1^	0.15	0.5
TIBC umol·L^−1^	0.24	0.3
Iron % saturation of TIBC	0.31	0.2
Ferritin µg·L^−1^	0.4	0.1
Alcohol units/week	−0.17	0.5
A1AT g·L^−1^	−0.22	0.3

BMI: body mass index, NEFA: non-esterified fatty acids, HOMA-S%: homeostatic model assessment—insulin sensitivity, HOMA-B%: homeostatic model assessment—β cell function, TIBC: total iron binding capacity, A1AT: α-1 antitrypsin.

**Table 2:** Baseline characteristics of study participants.

Population Characteristics	Median (Range)	Reference Range
Gender (*n* = 11)	10 males/1 female	-
Age	51 (32–67)	-
BMI kg·m^−2^	29.6 (20.2–40.4)	<25% *
Alcohol (units)	3 (Male)	0–24
	5 (Female)	0–14
TC mmol·L^−1^	5.7 (4.6–8.5)	<4.0 *
HDL mmol·L^−1^	Female 1.26	Female > 1.2 *
	Male 1.34 (0.2–1.49)	Male > 1.0 *
TG mmol·L^−1^	2.7 (0.6–6.0)	<1.7 *
NEFA umol·L^−1^	302 (138–491)	130–1050
Glycerol umol·L^−1^	90 (10–210)	27–137
Insulin mU·L^−1^	17.2 (8.3–87.4)	3.4–6.4 **
Glucose mmol·L^−1^	5.6 (5.0–7.5)	<6.1
HOMA-S%	43.9 (13.3–91.9)	100%
HOMA-B%	126.4 (92.6–254.5)	100%
Liver fat g·kg^−1^ water	44.0 (10.0–332.0)	<5.6 (95th centile)
ALT U·L^−1^	56 (19–119)	5–40
Iron umol·L^−1^	20.2 (10.2–28.1)	7–29
TIBC umol·L^−1^	65 (50–74)	45–70
Iron % of TIBC	33 (17.6–49.4)	<50% ***
Ferritin µg·L^−1^	187 (41.4–549.7)	15–200
Caeruloplasmin g·L^−1^	0.31 (0.2–0.39)	0.25–0.63
A1AT g·L^−1^	1.32 (1.07–1.95)	1.0–2.0

BMI: body mass index; TC: total cholesterol; HDL: high density cholesterol; TG: triglyceride; NEFA: non-esterified fatty acids; HOMA-S%: homeostatic model assessment—insulin sensitivity; HOMA-B%: homeostatic model assessment—β cell function; ALT: alanine transaminase; TIBC: total iron binding capacity; A1AT: α-1 antitrypsin; Reference ranges are 95th % confidence intervals unless otherwise indicated. * Clinic target levels; ** Interquartile range; *** British Society for Haematology Guideline 2000 on screening for haemochromatosis.
